# 

**Published:** 1917-11-17

**Authors:** 


### CONTENTS.

PAGE

**The Causes of Disease** ... ... ... ... 129

**War Emergency Medicines** ... ... ... 130

**Hospital and Institutional News** ... ... ... 131

The Royal Free's Appeal—Anti-research Propaganda—Measles on Board Ship—An Epigram on Salonika—Anti-typhoid Inoculation in France—A Sailors' Fund Sunday?—A Dangerous Competitor?—Should the Red Cross Supplement the Government Grant?—Sectional Subscriptions in Glasgow—War Service of Health Officials—British Red Cross in Italy—A Millionaire's Mansion for Wounded Americans—A Plague of Pension Committees—The Sugar-vouchers and Hospitals—American Women Doctors—Why Blanket Rationing is Imperative—Memorial to Dr. W. A. Haslam—A Beloved Hospital Commandant—A Proud Family Record—Educative Rest Clubs for Soldiers and Nurses—The Treatment of Venereal Diseases in Nottinghamshire—A Disputed Point—The Prince of Wales' Hospital, Cardiff—The *Franconia* Nursing Sisters—The Matron-Inspector—This Week's Drug Market—To Our Readers.

**Camp Disease on the Battle Fronts** ... ... 137

Victories of the Doctor and the Sanitarian.

**From Across the Seas** ... ... ... ... 138

**Payments for Patients in Voluntary Hospitals** 139

Treatment of War Pensioners and Others.

PAGE

**Parliament and the Profession** ... ... ... 141

**The Editor's Letter-Box** ... ... ... ... 142

The R.B.N.A. and the College.
King George's Fund for Sailors.

**The Burke Relief Foundation, New York** ... 143

The Physical Side of Social Regeneration.

**Nursing Affairs in Ireland** ... ... ... ... 144

The Value of Registration.

**The Matrons' and Sisters' Department** ... 145

The Nightingale School and St. Thomas's Hospital—II.
Round the Hospitals.
The Nation and Its Nurses—General Sir Alfred Keogh's Testimony—The British Press United to Help—British Families' Christmas Offering—Twenty-two Million American Children to Join and Help the Red Cross.

**Sister-Matron Katharine Monk** ... ... ... 147

Reredos in King's College Hospital Chapel.

**The League of National Safety** ... ... ... 150

Facts for Food Economists.

**Hospital and Institutional Results** ... ... 151

**King George's Fund for Sailors** ... ... ... 151

**Matrons' and Sisters' Appointments** ... ... 152

**The Institutional Worker** ... ... (Suppl.) 1

Starting a Venereal Block.

